# Transposable Element Is Predictive of Chemotherapy- and Immunotherapy-Related Overall Survival in Glioma

**DOI:** 10.3390/biomedicines13051177

**Published:** 2025-05-12

**Authors:** Bi Peng, Fan Shen, Ziqi Chen, Yongkai Yu, Rundong Liu, Yiling Zhang, Guoxian Long, Guangyuan Hu, Yuanhui Liu

**Affiliations:** 1 Department of Oncology, Tongji Hospital, Tongji Medical College, Huazhong University of Science and Technology, Wuhan 430030, Chinaoncolong@163.com (G.L.); 2 Nursing Department, Tongji Hospital, Tongji Medical College, Huazhong University of Science and Technology, Wuhan 430030, China; 3 Department of Dermatology, The First Affiliated Hospital of Nanjing Medical University, Nanjing 210000, China; 4 Cancer Center, Tongji Hospital, Tongji Medical College, Huazhong University of Science and Technology, Wuhan 430030, China

**Keywords:** transposable elements, overall survival, glioma

## Abstract

**Background:** Glioma is the most common type of malignant brain tumor. Temozolomide (TMZ) is a limited systematic treatment option for glioma, including low-grade glioma (LGG) and glioblastoma (GBM). However, not all patients benefit from TMZ and some develop resistance to it. MGMT methylation has been used to identify patients who may benefit from TMZ, but it is not effective in all cases. **Objectives:** There is an urgent need for new biomarkers to predict the survival of patients who receive TMZ. **Methods:** We utilized a recently developed method called REdiscoverTE to precisely measure the expression of transposable elements (TE). We performed Cox regression analysis to assess the predictive ability for prognosis and conducted a series of correlation studies to uncover potential mechanisms. **Results:** We identified three TEs, LTR81B, LTR27B, and MER39B, that were strongly predictive of longer survival in glioma patients receiving chemotherapy. We discovered that the expression of these TEs was positively associated with immune cells that enhance the immune system and negatively associated with immune cells suppressing the immune response, as well as molecules that control immune checkpoints. These three TEs were also found to predict better survival in patients receiving immunotherapy. **Conclusions:** In conclusion, we demonstrate that the expression of TEs can serve as a novel biomarker for the overall survival of glioma patients who receive TMZ chemotherapy or immunotherapy.

## 1. Introduction

Gliomas make up 30% of all brain tumors [1]. Grade 1 glioma is usually benign with favorable long-term outcomes from surgical resection. Grades 2 and 3 gliomas are commonly referred to as low-grade gliomas (LGG), while Grade 4 glioma is the most severe and known as glioblastoma (GBM). GBM is still a deadly condition despite advancements in treatments. It accounts for 80% of malignant brain tumors and is one of the most lethal forms of cancer. The average survival time is 12.2 to 18.2 months [2,3,4,5].

Temozolomide (TMZ) is a DNA alkylating agent that is currently the primary chemotherapy administered to GBM patients as it has transient tumor growth-arrest properties [6]. However, the use of TMZ in LGG is disputed and only the patients with high risk is widely recommended for TMZ chemotherapy [7,8]. Although the methylation status of the O6-methylguanine-DNA methyltransferase (MGMT) promoter is considered a useful biomarker for predicting outcomes and guiding treatment decisions in glioma, its actual value in this regard is still a subject of debate. The reason for discrepancies between MGMT promoter methylation status and treatment response in certain patients may be due to inconsistencies between MGMT methylation and expression levels in glioma [9]. Therefore, it is crucial to find a more accurate biomarker for predicting the efficacy of TMZ and the survival of patients treated with TMZ.

Transposable elements (TEs) comprise around half of the human genome [10], which are also known as “jumping genes” because of their ability to change their position within the genome and insert themselves into different positions. TEs are divided into two main classes based on their transposition mechanism, and these classes are further divided into subclasses, families, and over 1000 subfamilies [11]. Class I consists of retrotransposons, which use RNA as an intermediate to reverse-transcribe into cDNA and then insert themselves into other locations in the genome. Retrotransposons include Long Terminal Repeat (LTR) and non-LTR (LINE and SINE) elements. Class II consists of DNA transposons, which use either a “cut and paste” or “peel and paste” mechanism to change their genomic positions. Most TEs are rendered inactive through processes such as epigenetic silencing, point mutations, inverted rearrangements, and so on [12].

The relevance of TE expression to tumor immunity and immune response has been demonstrated across different types of cancers [13]. Additionally, studies have shown that TE is associated with the outcome of cancer patients in colorectal cancer [10], non-small cell lung cancer [14], and clear cell renal cell carcinoma [15]. However, the investigation of the usefulness of TE in predicting cancer treatment efficiency and survival in glioma has been very limited so far.

Here, we use a newly developed method called REdiscoverTE to accurately and reliably measure the expression of TEs using RNA-seq data. We have found that several TEs can effectively predict the clinical outcome of glioma, including GBM and LGG patients who have undergone chemotherapy. These TEs perform better than MGMT methylation and IDH mutation status in predicting outcomes. The prognostic TEs are associated with a decrease in DNA damage repair score and an increase in mTOR expression. Notably, these TEs are depleted in drug-tolerant persisters. Additionally, we have demonstrated that higher expression of TEs is linked to the presence of immune cells and predicts longer survival with immunotherapy.

## 2. Methods

### 2.1. Transposable Element Expression Quantification

We quantified TE subfamily expression from RNA sequencing data using the REdiscoverTE pipeline, as described by Kong et al. [13], which outperforms other approaches such as RepEnrich [16], SalmonTE [17], and the method by Rooney et al. [18]. This pipeline estimates read counts at the subfamily level without resolving individual genomic loci. Raw counts for each TE subfamily from REdiscoverTE were normalized using the RLE algorithm in edgeR, and log_2_CPM values were calculated with a prior count of 5 [19]. TE expression was categorized by genomic context—exonic, intronic, or intergenic—and the proportion of TE-derived reads was determined relative to total mapped reads (TEs + genes). We applied this method to multiple datasets (GSE48865, GSE121810, TCGA-GBM, and TCGA-LGG), focusing downstream analyses on the intergenic expression of 1059 TE subfamilies, classified into five major TE classes: long terminal repeats, DNA transposons, long interspersed nuclear element (LINEs), short interspersed nuclear element (SINEs), and retroposons.

### 2.2. Gene Expression Signatures and Score Calculation

The study compiled a total of 74 DNA damage repair signatures (21 from Reactome), 16 senescence signatures and 8 glycolysis signatures (Supplementary Table S1A–D) from curated databases, specifically MSigDB [20]. The signature scores across patient samples were calculated using gene set variation analysis (GSVA). To perform GSVA, the R package “GSVA (v1.42.0)” was utilized.

### 2.3. Cox-Ph Regression Survival Analysis

We used the Cox proportional hazards regression analysis with the ‘coxph’ function from the R ‘survival’ package (version 4.2.2) to determine the association between TE expression and overall survival. A z score less than −1.96 or greater than 1.96, which is equivalent to a *p*-value less than 0.05, was considered statistically significant and used as the threshold for determining favorable or unfavorable survival outcomes for each TE.

### 2.4. Evaluation of Immune Infiltration

To estimate the abundances of immune infiltrations in the tumor microenvironment, we applied Timer, Xcell, MCP-counter and cibersort to gene expression data [21,22,23,24]. The website tool (http://www.timer.cistrome.org) was used.

### 2.5. Correlations Studies

We analyzed the relation between expression of TEs with DNA damage repair signature scores, RPPA protein expression, immune infiltrates, immune checkpoint expression, senescence signature scores, glycolysis signature scores using the Pearson correlation test.

### 2.6. Sampling and Machine Learning

Survival models were trained and evaluated using a stratified, randomly shuffled outer cross-validation (CV) procedure, with a 75% training and 25% testing split, repeated 100 times and stratified by event status. Within each outer CV training set, hyperparameter tuning and model selection were performed by optimizing Harrell’s concordance index (C-index) using a stratified 4-fold inner CV, repeated five times. Model performance was assessed in both inner and outer CV loops by calculating C-index scores from predicted risk scores on held-out validation and test sets, respectively.

Clinical covariate-only survival models—including variables such as age at diagnosis, gender, grade, histology, MGMT methylation, and IDH mutation—were constructed using standard unpenalized Cox regression. In addition, four machine learning-based Cox models—Cox-Enet, Cox-RF, Cox-SVM, and Cox-Lasso—were built to jointly incorporate the 1059 TE subfamilies while adjusting for clinical covariates.

### 2.7. Cell Culture

Human U87 and U251 glioma cells were obtained from the Shanghai Institute of Cell Biology, Chinese Academy of Sciences (Shanghai, China). They were maintained in DMEM supplemented with 10% fetal bovine serum, 100 U/mL penicillin, and 100 μg/mL streptomycin at 37 °C in a humidified incubator with 5% CO_2_. Cells in the logarithmic growth phase were used for all experiments.

### 2.8. Drug-Tolerant Persister Assay and RNA Sequencing

U251 cells were treated with TMZ (600 μM) in combination with the mTOR inhibitor Torin1 (10 nM) for six days to induce drug-tolerant persisters, following the protocol established in our previous studies [25]. Four biological replicates of parental U251 cells and drug-tolerant persisters were subjected to RNA extraction using Trizol reagent (TIANGEN Biotech Co., Ltd., Beijing, China, Cat. No. DP424), followed by high-throughput RNA sequencing. Raw paired-end FASTQ reads were processed and quantified for TE expression using the REdiscoverTE pipeline, as described in Section 2.1.

### 2.9. CCK8 Drug Sensitivity Assay

U87 and U251 cells were seeded in 96-well plates at 5000 cells per well and incubated for 24 h. Experimental groups were treated with test compounds for 48 h, while control groups received PBS. After treatment, 10 μL of CCK-8 reagent (Dojindo Laboratories, Kumamoto, Japan) was added, and plates were incubated for 2 h. Optical density at 450 nm was measured, and cell viability was calculated as the normalized difference between treated and control wells, expressed as a percentage. All experiments were performed in triplicate, and statistical analysis was performed using GraphPad Prism 9.0.

### 2.10. Drug Synergy Score Calculation

Synergy analysis was performed using the SynergyFinder [26,27] online platform, with the Bliss independence model selected for evaluating the interaction between TMZ and decitabine (DAC) in vitro. The synergy score was calculated for each drug combination across varying concentrations. A synergy score less than −10 indicates antagonism, a score between −10 and 10 reflects an additive effect, and a score greater than 10 indicates a synergistic interaction.

### 2.11. Statistical Analysis

Associations between the expression of TEs and overall survival (OS) were analyzed using the Kaplan–Meier method. Survival curves were compared using the log-rank test. Statistical analysis for comparisons between two groups was conducted using the Wald test. The statistical software R (version 4.3.1) was used for all analyses, and *p*-values were calculated for both tails. A *p*-value less than 0.05 was considered statistically significant.

## 3. Results

### 3.1. The Prognostic Value of TE for OS in Glioma

**Figure 1:**
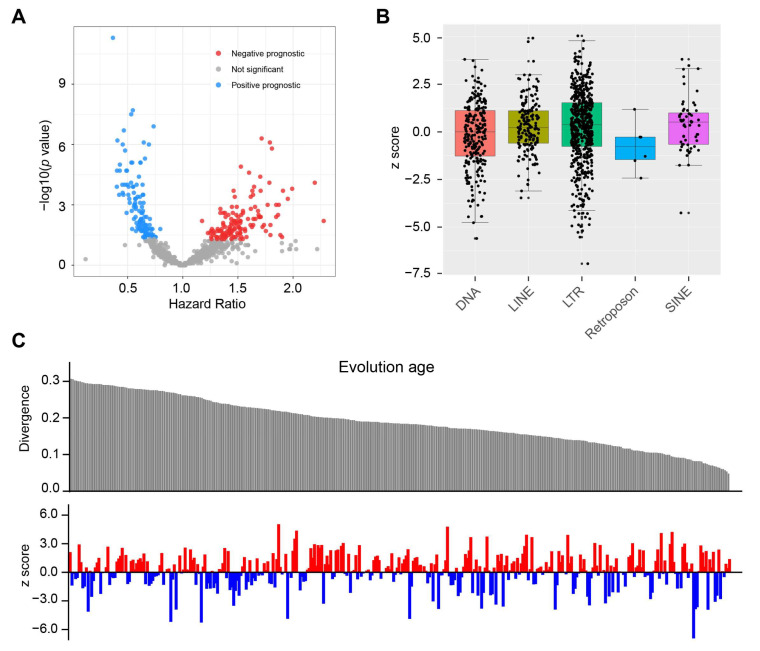
Prognostic landscape for 1059 TEs. (**A**) Volcano plot showing the calculated Cox regression models between TE’s expression and OS in the GSE48865 dataset. Hazard ratios were plotted against the negative log10 of the Cox *p*-value for the 1059 TEs found expressed in GSE48865. Significant HRs (*p* < 0.05) are colored. HR < 1 indicates an association between TE expression and improved OS; HR > 1 indicates an association between TE expression and worse OS. (**B**) Bar plot displaying the z scores of the Cox models for 1059 TEs covering five major TE families. A z score > 1.96 suggests that the presence or upregulation of a feature is associated with shorter survival times, while a z score < −1.96 suggests that the presence or upregulation of a feature is associated with longer survival times. (**C**) Correlation of evolution age reflected by divergence score with the prognostic value of TEs indicated by z score. The higher divergence score confers a younger evolution age. The red color denotes a predicted poor prognosis, whereas blue indicates favorable survival outcomes.

To investigate the landscape of TE expression in glioma, we initially measured the expression of 1059 TEs at the subfamily level in the GSE48865 cohort. To identify the chemotherapy-related survival, we performed a Cox survival analysis for the patients who have received chemotherapy. This analysis identified 263 TEs that had significant prognostic value (*p* < 0.05, Figure 1A). For each Cox model, we calculated z scores, which indicate the direction and significance of the relationship between survival [28,29]. A z score > 1.96 suggests that the presence or upregulation of a feature is associated with shorter survival times, while a z score < −1.96 suggests that the presence or upregulation of a feature is associated with longer survival times. We examined the prognostic power of TEs in each family (Figure 1B). The LTR and DNA families had the highest percentage (29% and 24%, respectively) of significant prognostic TEs (Supplementary Figure S1A). We then assessed the evolutionary age of TEs using divergence score and examined its association with prognostic ability (Figure 1C).

### 3.2. TEs Perform Better than IDH and MGMT for Predicting OS

**Figure 2:**
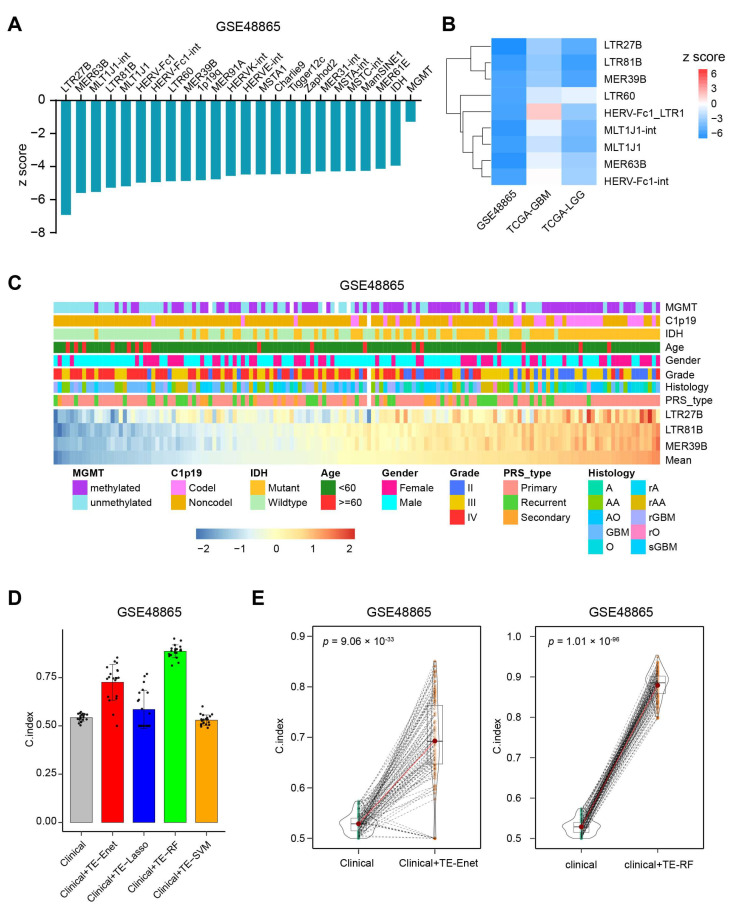
TEs perform better than clinical variates for predicting overall survival. (**A**) The 22 most prognostic TEs for the favorable outcome that outperform IDH and MGMT. (**B**) The validation of prognostic ability of the 10 most favorably prognostic TEs from GSE48865 in TCGA-GBM and TCGA-LGG datasets. (**C**) The landscape of clinicopathological features correlated with the expression of the three most prognostic TEs. The bottom heatmap displayed the expression profiles of the three most prognostic TEs as ordered by the mean of these three TEs. The TEs are annotated with MGMT methylation, C1p19 deletion, IDH mutation, age, gender, grade, histology and PRS type. Columns represent patients. (**D**) Data are presented as mean values ± standard deviation of the mean (SDM) for 20 random sampling split, as described in the methods section. C-index scores were obtained from multi-variate Cox models (clinical) and Cox-Enet, Cox-Lasso, Cox-RF, Cox-SVM models (clinical + TE) in GSE48865. (**E**) C-index score violin density plots for *n* = 100 sampling splits for the multi-variate Cox models (clinical) and Cox-Enet (left panel), Cox-RF (right panel) models.

It has been established that MGMT methylation and IDH mutation are important factors in determining sensitivity to TMZ chemotherapy [30]. We wanted to investigate whether TE is a better predictor of TMZ chemotherapy-related survival compared to MGMT methylation and IDH mutation status. Interestingly, it was found that 22 TEs analyzed performed better than MGMT methylation and IDH mutation based on the z score of Cox models (Figure 2A). To further validate these findings, Cox models were created using TCGA-GBM and TCGA-LGG datasets (Supplementary Table S2). Among the 22 TEs, three of them, namely LTR27B, LTR81B, and MER39B, consistently showed prognostic predictive ability (Figure 2B). Additionally, patients with different expression levels of LTR27B, LTR81B, and MER39B exhibited similar patterns of clinical characteristics (Figure 2C).

In order to evaluate the added value of TEs in the OS prediction, we built four different kinds of Cox machine learning models, including Cox-Enet, Cox-RF, Cox-SVM and Cox-Lasso, which jointly include the 1059 TE while simultaneously being able to control for prognostic clinical covariates, such as age at diagnosis, gender, grade, histology, MGMT methylation, IDH mutation. Additionally, we built standard Cox regression models using only the clinical factors. We evaluated the predictive performance of our models using Harrell’s concordance index (C-index), which measures the accuracy of survival model predictions. Each model analysis produced 100 instances, and C-index scores were obtained by randomly shuffling the data. Our results showed that the models combining clinical factors and TEs performed better than the models using clinical factors alone in the GSE48865 dataset (Figure 2D,E, Supplementary Figure S1B). Furthermore, the additional prognostic value of TEs was confirmed in the TCGA-GBM and TCGA-LGG datasets (Supplementary Figure S1C).

### 3.3. Prognostic TEs Are Correlated with Decreased DNA Damage Repair, Increased mTOR Activity and Improved Drug Sensitivity

**Figure 3:**
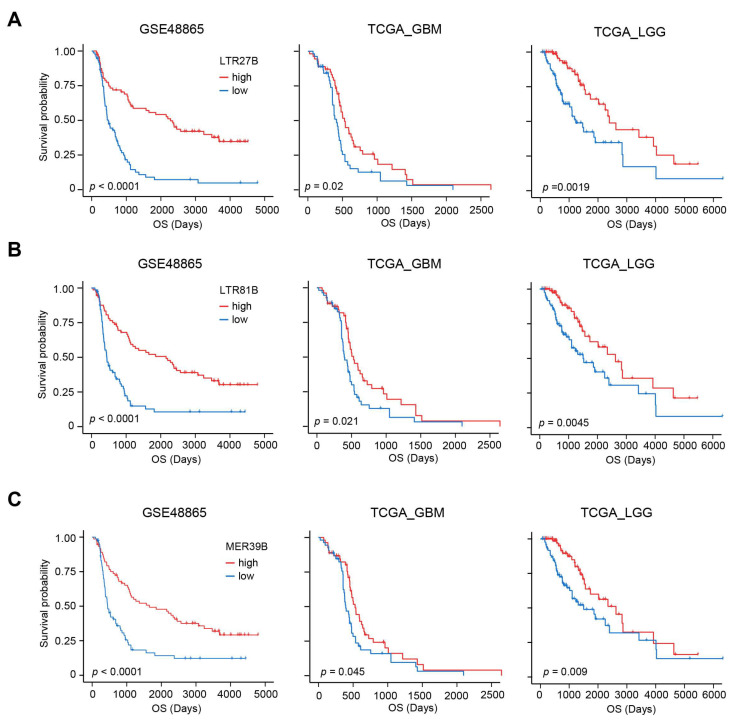
Expression of LTR27B, LTR81B and MER39B predicts favorable chemotherapy-related survival. (**A**) Kaplan–Meier plots displaying the prognostic ability of LTR27B for OS prediction in GSE48865, TCGA-GBM and TCGA-LGG datasets. (**B**) Prognostic value of LTR81B with Kaplan–Meier survival analysis for OS in GSE48865, TCGA-GBM and TCGA-LGG datasets. (**C**) Kaplan–Meier curves with patients stratified according to MER39B expression (low vs. high) for OS in GSE48865, TCGA-GBM and TCGA-LGG datasets.

**Figure 4:**
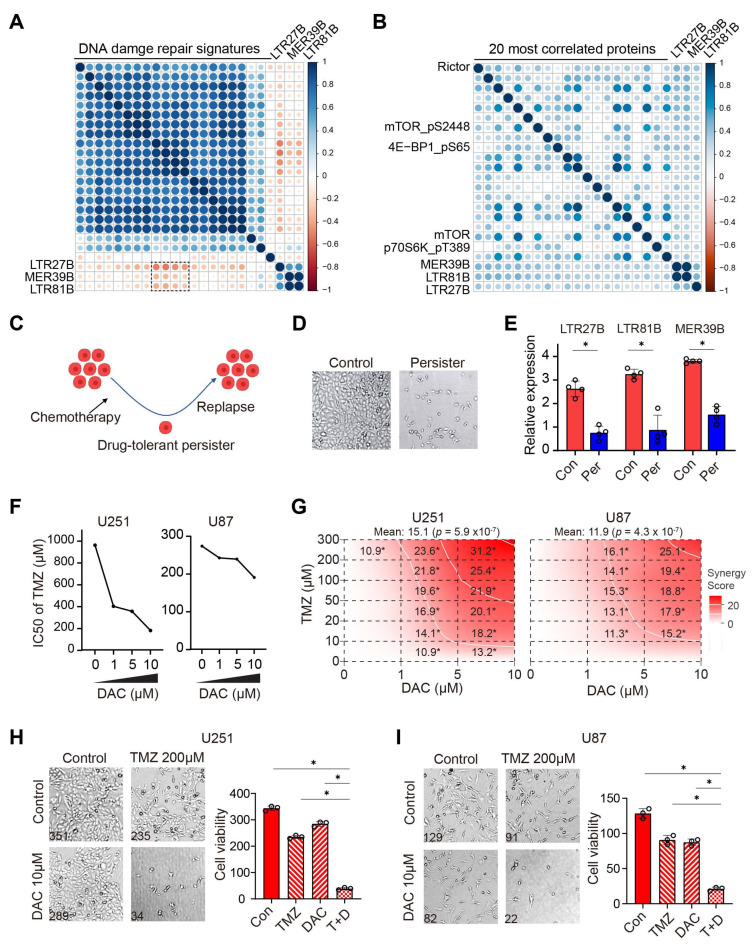
Higher expression of LTR27B, LTR81B, and MER39B associated with suppressed DDR, activated mTOR signaling and increased chemotherapy response. (**A**) Bubble matrix showing the correlation between 21 Reactome DNA damage repair signatures score and expression of the three identified TEs LTR27B, LTR81B and MER39B. (**B**) Bubble matrix representing the correlation between RPPA protein expression level and expression of LTR27B, LTR81B and MER39B. (**C**,**D**) Schema (**C**) and representative image (**D**) of drug-tolerant persister (DTP) induction. U251 cells were treated with TMZ in combination with an mTOR inhibitor Torin1, and both pre-treated control cells and DTP cells were collected for RNA sequencing. (**E**) LTR27B, LTR81B and MER39B expression in control (con) and persister (per) cells from RNA sequencing results. * *p* < 0.05. (**F**) IC50 values of TMZ with or without decitabine (DAC) measured by CCK8 assay in U251 and U81 cells. (**G**) Synergy score of TMZ and DAC combination treatment in U251 and U81 cells. * *p* < 0.05. A synergy score between −10 and 10 reflects an additive effect, and a score greater than 10 indicates a synergistic interaction. (**H**,**I**) Representative images and quantification of TMZ and DAC combination treatment in U251 (**H**) and U81 (**I**) cells. * *p* < 0.05.

After establishing that higher expression of LTR27B, LTR81B, or MER39B is associated with longer overall survival in chemotherapy (Figure 3A–C), we investigated the relationship between TE and other features to explore the possible mechanism. It has been reported that DNA damage repair (DDR) plays a significant role in determining the effectiveness of chemotherapy [31,32]. We conducted a systematic analysis of DDR gene signatures using the GSEA database and identified 74 distinct signatures (Supplementary Table S1). We quantified GSVA scores for these 74 DDR signatures for each patient and calculated the correlation the correlation between the expression of LTR27B, LTR81B, or MER39B and DDR scores. Interestingly, we found that the expression of LTR27B, LTR81B, or MER39B was mostly negatively correlated with DDR scores (Figure 4A), further supporting the role of these TEs in predicting chemotherapy-related survival, as we demonstrated earlier.

To gain insight into the potential mechanisms underlying the correlation between expression of LTR27B, LTR81B, or MER39B and improved survival, we utilized protein expression RPPA data to systematically examine the proteins associated with these three TEs. We found that Rictor, a component of the mTOR complex, is the protein most strongly correlated with the three TEs (Figure 4B). Additionally, mTOR, mTOR-pS2448, and its downstream phosphorylated forms, including 4EBP1-pS65 and p70S6K-pT389, were also found to be positively correlated with the three TEs. These results are consistent with our previous findings that inhibiting mTOR leads to chemotherapy resistance, while activating mTOR improves drug efficacy [25]. To validate our findings, we treated the GBM cell line U251 with TMZ chemotherapy in combination with mTOR inhibition, inducing drug-tolerant persisters (Figure 4C,D), in a manner similar to the methods used in our previous study [25]. The expression of LTR27B, LTR81B, and MER39B was markedly decreased in persister cells compared to control cells (Figure 4E), reinforcing the positive correlation between their expression and enhanced sensitivity to TMZ.

Given that mTOR inhibition is associated with cellular senescence [25], we examined the correlation between the three TEs and sixteen senescence signatures. Nine of the sixteen signatures, including “Leukotriene metabolism senescence”, “Metabolism in oncogene-induced senescence”, and “Glycolysis in senescence”, were significantly negatively correlated with TE expression (Supplementary Figure S2A). Noting that many of these signatures are metabolism-related, particularly glycolysis, we further compared TE expression with eight glycolysis signatures. The three TEs showed a negative correlation with nearly all glycolysis signatures, suggesting a potential link to glucose metabolism, which may underlie their role in chemotherapy sensitization and warrants further investigation (Supplementary Figure S2B).

Since DNA methyltransferase inhibitors (DNMTis) such as decitabine (DAC) have been shown to reactivate TE expression [10,33], we evaluated the effect of TE activation on TMZ sensitivity by treating GBM U251 and U87 cells with DAC. The addition of DAC sensitized the cells to TMZ, as evidenced by a decreased IC50 of TMZ (Figure 4F). To further evaluate the combinatorial effect, we employed SynergyFinder (version 3.0) [25,26], a computational tool designed to quantify drug interactions. Notably, the combination therapy exhibited robust synergy, with scores exceeding the threshold of 10 in most tested conditions (*p* < 0.05; Figure 4G), providing compelling evidence for DAC-TMZ synergism (Figure 4H,I).

### 3.4. TEs Expression Is Linked with Immune Modulation and Predicts Immunotherapy Outcome

**Figure 5:**
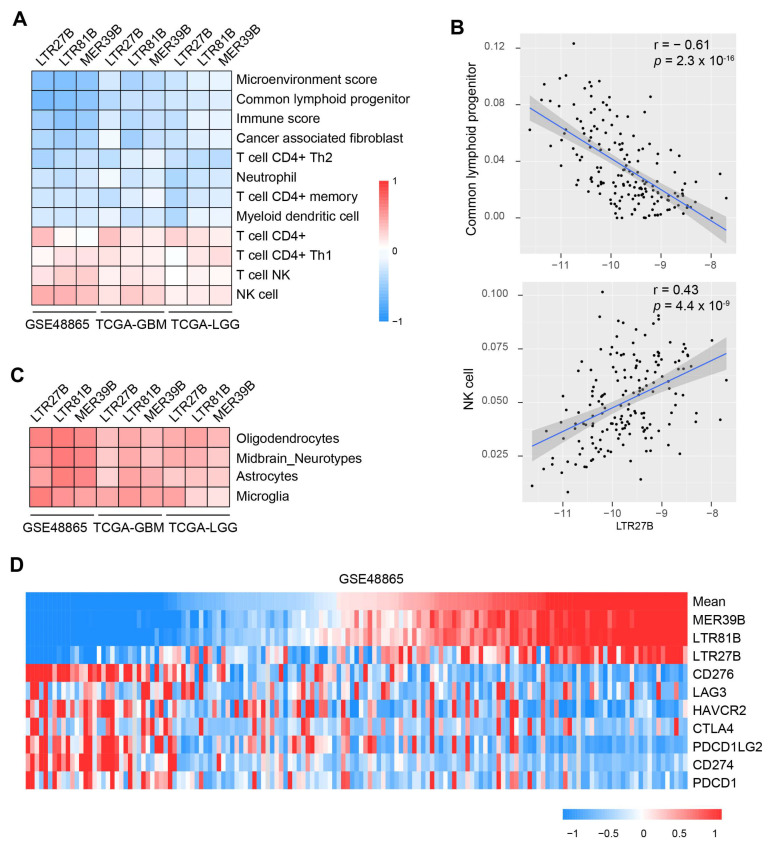
Relationship between TEs expression and immune infiltrates and immune checkpoints. (**A**) Heat map showing the immune infiltrates that are significantly correlated with LTR27B, LTR81B and MER39B across the datasets GSE48865, TCGA-GBM and TCGA-LGG. (**B**) Correlation of LTR27B expression and common lymphoid progenitor (top panel) or NK cell (bottom panel). (**C**) Heat map showing the oligodendrocytes, midbrain-neurotypes, astrocytes, microglia that are positively correlated with LTR27B, LTR81B and MER39B expression across the TEs LTR27B, LTR81B and MER39B. (**D**). Heat map showing the expression of LTR27B, LTR81B, MER39B compared with the expression of immune inhibitory checkpoint proteins.

As immunogenic cell death is one of the major mechanisms of cytotoxic chemotherapy, and the combination of chemotherapy and immunotherapy has been increasingly studied, we investigated the relationship between TEs and immune infiltrates and modulators. We used the Timer and Xcell methods to obtain the immune infiltrates. An unbiased Pearson’s correlation analysis revealed a reverse relationship between TE expression and inhibitory immune cells, such as common lymphoid progenitor, cancer-associated fibroblast, neutrophil, and myeloid dendritic cell (Figure 5A,B). On the other hand, we observed a positive correlation between TE expression and stimulatory immune infiltrates, including NK cells and CD4+ T cells. We also found a positive correlation between TEs and immune cells specific to the neurosystem, such as oligodendrocytes, astrocytes, and microglia (Figure 5C). Additionally, we found that patients with lower expression of immune checkpoint molecules (such as CD276, CD274, PDCD1) had higher levels of LTR27B, LTR81B, and MER39B (Figure 5D). These results suggest that immune stimulatory factors may contribute to the beneficial role of TEs in chemotherapy-related survival.

**Figure 6:**
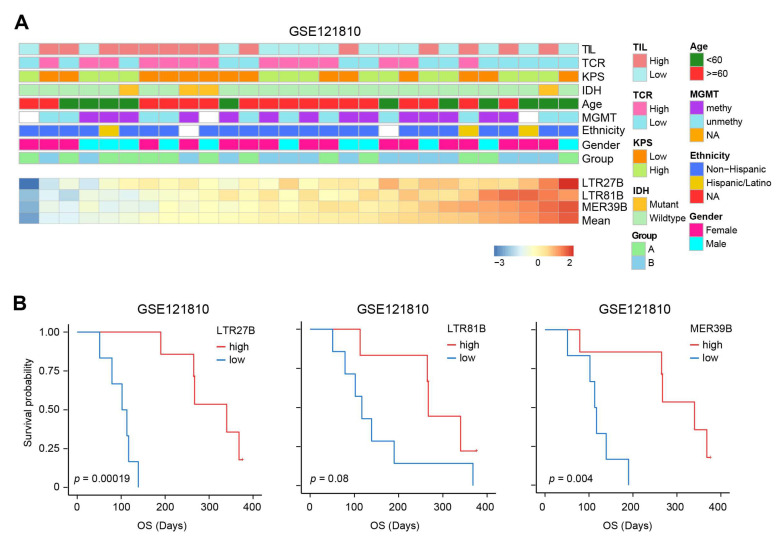
Higher expression of LTR27B, LTR81B, and MER39B confer better immunotherapy-related survival. (**A**) The landscape of clinicopathological features correlated with expression of the three most prognostic TEs in GSE121810. The bottom heatmap displayed the expression profiles of the three most prognostic TEs as ordered by the mean of these three TEs. The TEs are annotated with tumor infiltrated leukocytes (TIL), TCR, KPS, IDH mutation, age, MGMT methylation, ethnicity, gender, grade and treatment group. The columns represent patients. (**B**) Kaplan–Meier plots displaying the prognostic ability of LTR27B, LTR81B and MER39B for OS prediction in the GSE121810 immunotherapy dataset.

To further investigate the clinical relevance of TEs in immunotherapy, we examined the expression of three TEs (LTR27B, LTR81B, MER39B) in a dataset of patients who received immunotherapy. In dataset GSE121810, patients with different levels of TE expression showed similar patterns of clinical characteristics (Figure 6A). The expression levels of LTR27B and MER39B can significantly predict improved survival for patients who received adjuvant immunotherapy (Figure 6B). The higher expression of LTR81B also predicts better survival, though it does not reach a significant extent. These findings suggest that TEs, particularly LTR27B and MER39B, may serve as potential biomarkers for predicting the effectiveness of immunotherapy in glioma patients.

## 4. Discussion

In this study, we demonstrate for the first time the use of TEs as biomarkers and predictors for chemotherapy and immunotherapy-related survival in glioma. We also showed the correlation between TE expression and DNA damage repair score and mTOR expression. Additionally, we validated the association between TE expression and drug-tolerant persister induction. Furthermore, we revealed that the expression of TEs links immune infiltrates and immune modulators. These findings provide novel insights into the role of TEs in predicting survival and new possibilities to achieve accurate personalized systematic treatment for glioma.

Though TE have been focused on for long time, it is challenging and complicated by uncertainty in fragment assignment. To address this issue, we used the recent software REdiscoverTE (version 1.0). This software provides accurate estimation of ERV expression resolved to specific genomic locations. In this study, we split the patients who have received chemotherapy to perform the Cox regression analysis and find the chemotherapy-specific survival predictors. This distinction of patient selection can reduce the confounding factor caused by the patients who did not receive chemotherapy. Furthermore, compared to the studies of the drug response, the study of overall survival is more crucial for patients as it represents long-term clinical benefits. Additionally, overall survival has the advantage of being less ambiguous than drug response, as it clearly defines whether a patient is alive or deceased.

We have identified three TEs, LTR81B, LTR27B, MER39B, that significantly predict good survival for patients who have received TMZ chemotherapy across three datasets. LTR27B and MER39B belong to the ERV1 family, while LTR81B belongs to the Gypsy family. And all of these three TEs belong to the LTR class. An inverse correlation between the expression of the three identified TEs and the DDR score was found. And decreased DDR signature scores may contribute to a good chemotherapy response, resulting in better survival [34]. Additionally, we demonstrated that the expression levels of LTR81B, LTR27B, and MER39B were markedly decreased in drug-tolerant persister cells, further supporting their role in chemotherapy sensitization. We also showed that treatment with the DNA methyltransferase inhibitor DAC enhanced TMZ sensitivity, highlighting that TE activation may represent a viable strategy to augment chemotherapy efficacy and improve outcomes for glioma patients.

Recently, it has been shown that some TEs, such as LTR and especially HERVs, can serve as tumor antigen signals [35]. The transient HERVs expression may derive peptides and induce an innate immune response [36]. A thorough examination of HERVs on immune infiltrates and modulators in brain neoplasms, specifically glioma, has yet to be performed. Here, we noted that the three most prognostic TEs (LTR81B, LTR27B, MER39B), which all belong to the LTR class, are positively related to the stimulatory immune cells. In contrast, the expression of LTR81B, LTR27B, MER39B are negatively related to the abundance of inhibitory immune cells and expression of immune checkpoints. All these findings may constitute the mechanism for the positive relationship between the expression of LTR81B, LTR27B, MER39B and better immunotherapy survival. Specifically, while higher LTR81B expression predicts better survival, statistical significance was not reached, likely due to the limited sample size (only 29 patients). To address this limitation and further clarify the role of LTR elements in glioma immunology, future work will focus on expanding cohort sizes, performing mechanistic studies to dissect how LTR expression modulates the immune microenvironment, and exploring the therapeutic potential of targeting or harnessing LTRs to improve immunotherapy efficacy.

TEs are typically silenced in the genome through mechanisms such as epigenetic repression, point mutations, and structural rearrangements. However, the genomic instability and epigenetic dysregulation associated with cancer can compromise these silencing mechanisms, leading to aberrant TE reactivation. Consequently, the level of TE expression may serve as an indirect marker of the extent of oncogenic disruption, making TEs potential predictors of tumor aggressiveness or malignancy. It supports the use of TE expression as a biomarker not just for prognosis but potentially for tumor grading or therapy stratification. TE expression may reflect genomic chaos rather than actively contributing to tumor biology. Thus, they may be a marker rather than a mechanistic driver. To clarify this, functional validation studies are warranted. Interestingly, many non-cancerous conditions, including inflammation, aging and neurodegeneration, also affect epigenetic silencing and might lead to TE expression. This could limit the specificity of TEs as cancer-specific markers. For example, some TEs are markedly overexpressed in epilepsy [37]. A more thorough investigation to distinguish the utility of TEs as markers in cancer versus non-cancerous illness is thus required.

## 5. Limitations

We have to acknowledge the limitation that this study is primarily correlational. Although the correlations are robust and multi-dimensional, and we have provided experimental validation showing decreased expression of the identified TEs in drug-tolerant persisters and strong synergy between TE expression inducers and chemotherapy, further in-depth and comprehensive functional experiments (e.g., in vitro modulation of TE expression) are necessary to better understand the potential causal role of TEs.

## 6. Conclusions

To conclude, our study demonstrated that the TEs, which had been previously ‘hidden’ to RNA-seq studies, contained features strongly correlated with the survival of glioma patients who received chemotherapy or immunotherapy. It also provides evidence that the addition of TEs to clinical features could be used to largely improve survival prediction.

## Data Availability

The RNA-seq datasets were retrieved from Gene Expression Omnibus (GEO) with the accession numbers: GSE48865 and GSE121810. The TCGA-GBM and TCGA-LGG datasets were obtained from cBioPortal site (https://www.cbioportal.org/, accessed on 1 January 2024). Any other relevant data can be found in the article, supplementary information, or can be requested from the corresponding author.

## Supplementary Materials

The following supporting information can be downloaded at: https://www.mdpi.com/article/10.3390/biomedicines13051177/s1, Supplementary Figure S1. Performance comparison between clinical variates alone models and combinational clinical variates and TEs models. (A). C-index score violin density plots for n = 100 sampling splits for the multi-variate Cox models (clinical) and Cox-SVM (left panel), Cox-Lasso (right panel) models. (B). Data are presented as mean values ± standard deviation of the mean (SDM) for 20 random sampling split, as described in Methods. (C) index scores are obtained from multi-variate Cox models (clinical) and Cox-Enet, Cox-Lasso, Cox-RF, Cox-SVM models (clinical + TE) in TCGA-GBM (left) and TCGA-LGG (right). Supplementary Figure S2. Correlation between senescence signature scores, glycolysis signature scores and expression of LTR27B, LTR81B and MER39B. (A). Bubble matrix showing the correlation between 16 senescence-related signatures score and expression of the TEs LTR27B, LTR81B and MER39B. (B). Bubble matrix showing the correlation between 8 glycolysis-related signatures score and expression of the TEs LTR27B, LTR81B and MER39B. Supplementary Table S1. Gene signatures from the MSigDB database Used for GSVA Analysis. Supplementary Table S2. Demographic characteristics of the main datasets used in this study.
