# Supplementary figures and images for: Transposable Element Is Predictive of Chemotherapy- and Immunotherapy-Related Overall Survival in Glioma
Source: Biomedicines. 2025 May 12;13(5):1177. doi: 10.3390/biomedicines13051177 (PMC12109447; doi:10.3390/biomedicines13051177)

# Supplementary Figure S1

A

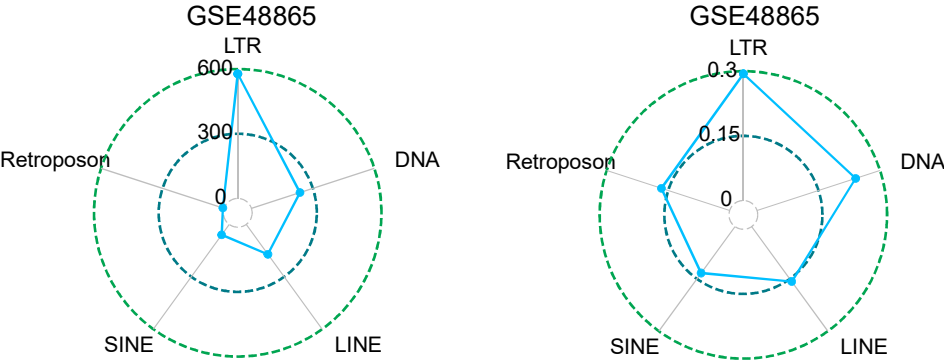

B

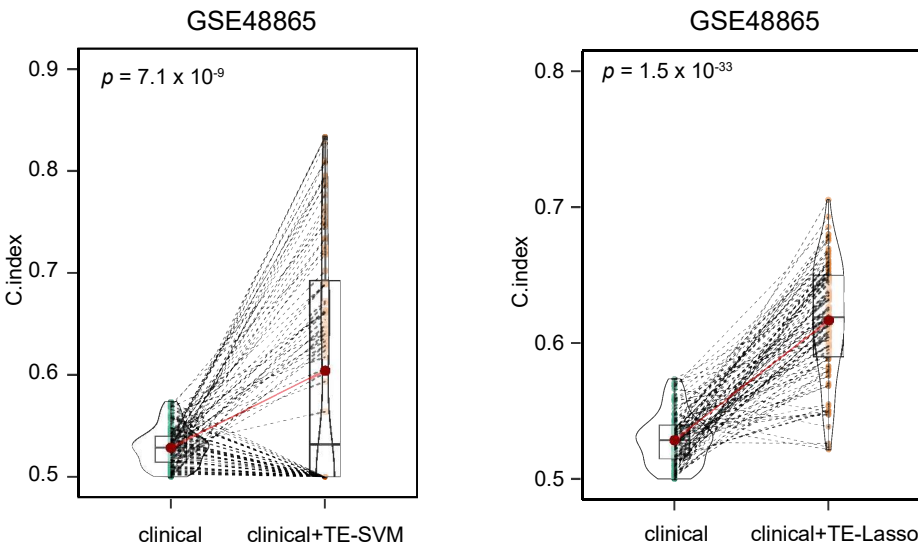

C

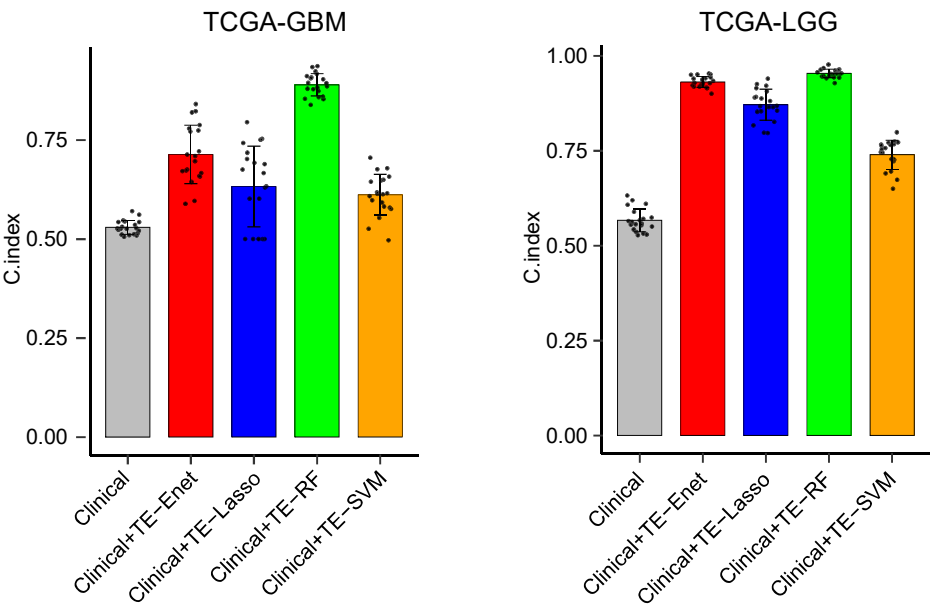

# Supplementray Figure S2

A

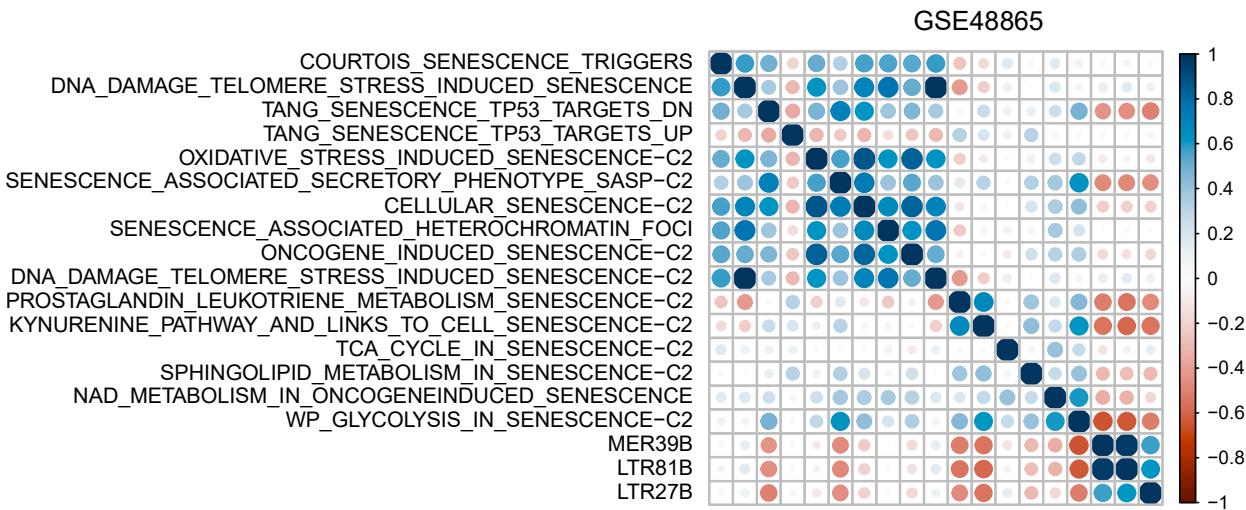

B

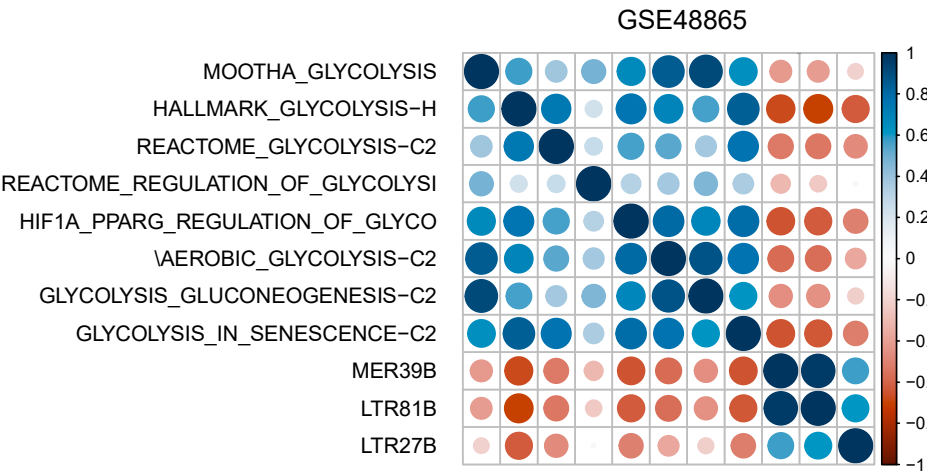

Supplement: Supplementary file 1 [file biomedicines-13-01177-s001.zip › biomedicines-3597848-supplementary-figures.pdf]
